# Data on groundwater quality, scaling potential and corrosiveness of water samples in Torbat-e-Heydariyeh rural drinking water resources, Khorasan-e-Razavi province, Iran

**DOI:** 10.1016/j.dib.2018.06.062

**Published:** 2018-06-26

**Authors:** A.A. Babaeia, Gh. Goudarzi, Rouhollah Khodadadi, Davoud Jalili, Majid Radfard, Hamed Biglari, Abbas Abasnia, Arefeh Mirzabeigi

**Affiliations:** a Environmental Technologies Research Center (ETRC), Ahvaz Jundishapur University of Medical Sciences, Ahvaz, Iran; b Associate Professor, Environmental Technologies Research Center, Ahvaz Jundishapur University of Medical Sciences, Ahvaz, Iran; c Department of Environmental Health Engineering, School of Health, Student Research Office, Shahid Beheshti University of Medical Sciences, Tehran, Iran; d Department of Environmental Health Engineering, School of Health, Ahvaz Jundishapur University of Medical Sciences, Ahvaz, Iran; e Department of Environmental Health Engineering, School of Health, Ahvaz Jundishapur University of Medical Sciences, Ahvaz, Iran; f Health deputy shahrekord University of Medical Sciences, shahrekord, Iran; g Health Research Center, Life Stayle institute, Baqiyatallah University of Medical Sciences, Tehran, Iran; h Department of Environmental Health Engineering, School of Public Health, Gonabad University of Medical Sciences, Gonabad, Iran; i Department of Environmental Health Engineering, School of Public Health, Tehran University of Medical Sciences, Tehran, Iran; j Department of Environmental Health Engineering, Bam University of Medical Sciences, Bam, Iran

**Keywords:** Drinking water, Villages of Torbat-e-Heydariyeh city, Stability index

## Abstract

According to World Health Organization guidelines, corrosion control is an important aspect of safe drinking-water supplies. The data presented is physical and chemical parameters of drinking water in the rural areas of Torbat-e-Heydariyeh city, also to determine corrosion indices. This cross-sectional study has carried out with 188 taken samples during 2014 with 13 parameters, which has been analyzed based on standard method. Also with regard to standard conditions, result of this paper is compared with Environmental Protection Agency and Iran national standards. Five indices, Langlier Saturation Index (LSI), Ryznar Stability Index (RSI), Puckorius Scaling Index (PSI), Larson-Skold Index (LS) and Aggressive Index (AI), programmed by using Microsoft Excel software. Owing to its simplicity*,* the program can easily be used by researchers and operators. Parameters included Sulfate, Sodium, Chloride, and Electrical Conductivity respectively was 13.5%, 28%, 10.5%, and 15% more than standard level. The amounts of Nitrate, in 98% of cases were in permissible limits and about 2% were more than standard level. Result of presented research indicate that water is corrosive at 10.6%, 89.4%, 87.2%, 59.6% and 14.9% of drinking water supply reservoirs, according to LSI, RSI, PSI, LS and AI, respectively.

**Specifications Table**

**Table:** 

Subject area	Chemistry
More specific subject area	Chemistry of groundwater
Type of data	Table and figure
How data was acquired	Experiments conducted in two general categories of device experiments and Titration. Titration Experiment includes temporary and permanent hardness, magnesium, calcium and chloride, Device Experiment consist of pH (model wtw, Esimmetrwb), Electrical conductivity, Turbidity (model Hach50161/co150model P2100Hach, USA), Fluorine, nitrate, sulfate
Data format	Raw, Analyzed
Experimental factors	188 samples from 47 water sources were taken, 18 parameters were evaluated according to the standard method, and compared with Iran and EPA water standards. Experiments conducted in two general categories of device experiments and Titration.
Experimental features	Titration Experiment includes temporary and permanent hardness, magnesium, calcium and chlorides, Device Experiment consist of pH, Electrical conductivity, Turbidity, Fluorine, nitrate, sulfate.
Data source location	Torbat-e-Heydariyeh, Razavi Khorasan Province, Iran
Data accessibility	Data are included in this article

**Value of the data**

• Determination of the physical and chemical parameter including EC, TDS, TH, CaH, pH, Turbidity, Cl^−^, NO_3_^−^, SO_4_^2^^−^, F, Na^+^ TDS, Ca^2+^, Mg^2+^, in ground water was investigated in rural area, Khorasan-e-Razavi province, Iran.
• Water distribution networks of many rural areas, requires attention to achieve the Iran quality standards of drinking water.
• Take the necessary actions in cases where water tends to be corrosive in the distribution network is necessary.

## 1 Data

**Fig. 1:**
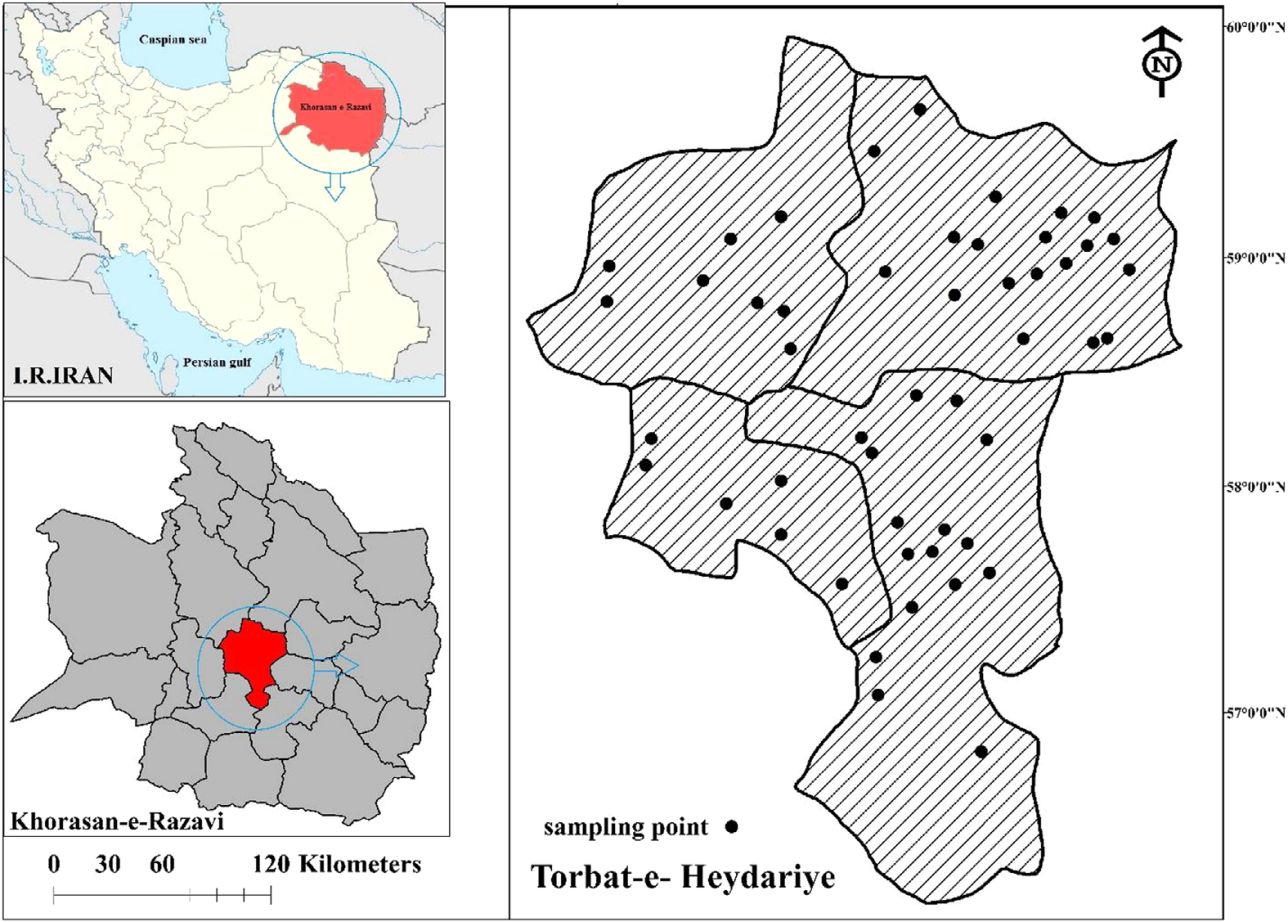
Location of water sampling sites in Torbat-e-Heydariyeh [1], [2].

Data presented here deal with monitoring of physical and chemical including EC, TDS, TH, CaH, pH, Turbidity, Cl, NO_3_^−^, SO_4_^2^^−^, F, Na^+^ TDS, Ca and Mg As in Khorasan-e-Razavi province, Iran. Fig. 1 shows location of water sampling sites in Torbat-e-Heydariyeh. Table 2 shows average of physical and chemical parameters of drinking water, water resources in the rural area of Torbat-e-Heydarie in 2014, Table 3 shows comparison drinking water resources in the rural area of Torbat-e-Heydarie in 2014, Table 4 shows calculation of water stability indices at sampling temperature.

**Table 1:** Indicators used in the study for distribution network from different rural of Torbat-e-Heydariyeh [1], [2], [3], [4], [5].

**Index**	**Equation**	**Index value**	**Water condition**
**Langelier Saturation Index**	LSI = pH-pHs	LSI > 0	Super saturated, tend to precipitate CaCO_3_
		LSI = 0	Saturated, CaCO_3_ is in equilibrium
		LSI < 0	Under saturated, tend to dissolve solid CaCO_3_
**Ryznar Stability Index**	RSI = pHs2-pH	RSI < 6	Super saturated, tend to precipitate CaCO_3_
		6 < RSI < 7	Saturated, CaCO_3_ is in equilibrium
		RSI > 7	Under saturated, tend to dissolve solid CaCO_3_
**Puckorius Scaling Index**	PSI = 2 (pHeq)–pHs	PSI < 6	scaling is unlikely to occur
	pH = 1.465 + log (T.ALK) + 4.54	PSI > 7	likely to dissolve scale
	pHeq = 1.465 × log(T.ALK) + 4.54		
**Larson-Skold Index**	Ls = (cl^−^ + SO_4_^2^^−^)/(HCO_3_^−^ + CO_3_^2^^−^)	LS < 0.8	Chloride and sulfate are unlikely to interfere with the formation of protecting film
		0.8 < LS < 1.2	Corrosion rates may be higher than expected
		LS > 1.2	High rates of localized corrosion may be expected.
**Aggressiveness Index**	AI = pH + log[(alk)(H)]	AI > 12	Non aggressive
		10 < AI < 12	Moderately aggressive
		AI < 10	Very aggressive

**Table 2:** Average of physical and chemical parameters of drinking water, water resources in the rural Area of Torbat Heydariyeh at 2014.

**Village**	EC (µMoh/cm)	TDS mg/l	T.H mg/l CaCO_3_	CaH mg/l CaCO_3_	pH	Turbidity NTU	Cl^−^mg/l	NO3^−^mg/l	SO4^2^^−^ mg/l	F^+^ mg/l	Na^+^ mg/l	Ca^2+^ mg/l	Mg^2+^ mg/l
Fath Abad	926	605	130	48	8.2	0.36	97	13	149	0.5	144	19	20
Seyuki	5250	3281	700	272	7.1	0.23	109	6	551	0.67	869	109	103
Senjed bour	848	530	130	96	7.9	0.68	153	8	23	0.22	124	38	8
Sanobar	225	141	82	60	8	0.20	4	6	9	0.08	17	24	5
Hesar	549	343	110	56	7.8	0.55	34	10	37	0.28	75	22	13
Kashkak	1305	816	460	396	7.7	0.01	70	7	338	0.59	101	158	15
Kameh Sofla	1406	879	364	224	7.8	0.28	214	11	128	0.22	173	90	34
Kame Olia	1004	628	310	162	7.8	0.43	137	7	65	0.21	104	68	36
Sorkhabad	1627	1017	368	236	7.5	0.54	160	17	213	0.54	218	94	32
Khoroshbar	1525	953	590	444	7.2	0.30	137	80	152	0.12	105	178	35
Besk	908	568	324	232	7.5	3.8	24	30	76	0.39	80	93	22
Fadihah	1070	669	296	184	7.8	4.95	81	14	82	0.43	123	74	27
Rud Majan	1004	628	364	200	7.5	0.58	116	14	26	0.24	83	80	39
Deh Paeen	1316	823	244	148	7.7	0.52	190	37	58	0.40	192	59	23
Fahandar	1542	964	224	144	7.9	0.31	291	37	73	0.47	264	58	19
Khorram	2730	1706	308	152	8.2	0.30	468	14	465	0.67	433	61	37
Deh Menar	1242	776	104	36	8.4	0.35	111	16	246	0.67	215	14	16
Abd Abad	1360	850	264	92	8.2	1.3	232	15	176	0.56	180	37	41
Bors	1122	701	292	160	7.8	0.25	140	7	218	0.28	119	64	32
Tajrud	1827	1142	180	100	8.1	0.44	250	7	316	0.51	320	40	19
Houze Sorkh	2760	1725	212	120	7.8	1.8	576	27	213	0.59	490	48	22
Seyuki	2300	1438	144	80	8	0.35	434	18	168	0.60	440	32	15
Bouri Abad	2590	1619	120	76	8.1	0.35	517	22	294	0.87	489	30	11
Kaj Derakht	769	481	124	100	7.8	0.70	77	25	78	0.41	13	40	6
Seyuki	2310	1444	152	84	8	0.70	454	18	194	0.66	411	34	16
Haji Beygi	1654	1034	544	380	7.6	0.52	191	13	312	0.39	150	152	39
Asadieh	878	549	100	40	8.4	0.78	98	14	100	0.27	148	16	14
Shileh Goshad	524	328	172	84	8.3	0.60	27	13	68	0.35	48	34	21
Asad Abad	1439	899	240	64	8.2	1.6	262	26	151	0.57	205	26	42
Heshmat Abad	1692	1058	284	120	8.3	1.5	148	14	485	0.38	235	48	39
Sarhang	1010	631	168	60	8.3	0.30	149	20	144	0.29	163	24	26
Kal Qari	1299	812	456	48	8.2	0.20	155	23	173	0.19	105	19	98
Pish Akhor	2600	1625	326	84	8.1	0.75	389	6	482	0.72	500	34	58
Soltan Abad	1448	905	200	60	8.3	0.44	223	12	219	0.55	230	24	34
Asfiukh	1674	1046	232	120	7.8	0.07	341	16	121	0.29	260	48	27
Robat Miandasht	1412	883	236	92	8.1	0.90	258	26	199	0.19	235	37	35
Nasar	1865	1166	356	84	8	0.08	388	9	206	0.29	265	34	65
Mahmoud Abad	1196	748	184	40	8.1	1.4	127	12	137	0.28	202	16	35
Asad Abad	1613	1008	352	92	8	2.2	290	26	236	0.30	232	37	62
Pangi	604	378	152	60	8.1	0.36	33	6	100	0.30	76	24	22
Esfiz	711	444	128	108	7.9	0.38	71	6	168	0.20	104	43	5
Razg	519	324	248	157	8	0.13	9	3	36	0.16	16	63	22
Nasar	260	163	64	52	8.2	0.23	18	6	32	0.08	28	21	3
Derakht Senjed	1477	923	72	24	8.3	0.21	16	7	188	0.43	300	10	12
Nouri	1624	1015	240	100	8.3	0.18	104	18	487	0.60	253	40	34
Sarbala	1350	844	132	32	8.3	0.14	88	11	463	0.63	237	13	24
Nosrat Abad	1288	805	100	32	7.9	0.31	111	16	266	0.60	238	13	16

**Table 3:** Comparison drinking water resources in the rural area of Torbat-e-Heydarie in 2014 [6], [7], [8], [9], [10], [11], [12].

		Iran national standard 1053		%villages			
**Parameter**	Unit	Maximum allowable	Minimum desirable	Higher	Optimum	Permissible	EPA standard MCL
PH	Dimensionless	6.5–9	6.5–8.5	–	%100	–	6.5–8.2
TDS	mg/l	1500	500	%21	%68	%11	500
Cl	mg/l	400	250	%15	%74.5	%10.5	
So4	mg/l	400	250	%10	%76.5	%13.5	250
No3	mg/l	50	–	%98	–	%2	10
Ca	mg/l	400	300	–	%100	–	
Mg	mg/l	150	30	%43	%75	–	
Na	mg/l	200	200	%21	%51	%28	
F	mg/l	1.5	0.5	%36	%64	–	2
T.H	mg/l(CaCO_3_)	500	200	%3.5	%30.5	%66	–
Tur	NTU	5	Less than 1	17	%83	–	1
EC	μmhos/cm	2000	1500	%21	%64	%15	–

**Table 4:** Calculation of water stability indices at sampling temperature.

Village	LSI	RSI	PSI	LS	AI	Village	LSI	RSI	PSI	LS	AI
Fathabad	0.15	7.95	7.91	1.08	11.839	Kaj Derakht	0.16	7.47	6.96	0.72	12.12
Seyuki	− 0.44	7.93	6.80	2.96	11.657	Seyuki	0.45	7.12	6.65	2.30	12.47
Senjed bour	0.07	7.82	7.57	1.41	12.149	Haji Beygi	0.69	6.22	5.33	1.36	12.68
Sanobar	− 0.14	8.33	8.33	0.09	11.878	Asadieh	0.33	7.76	7.88	0.95	12.33
Hesar	− 0.19	8.20	7.73	0.33	11.874	Shileh Goshad	0.51	7.29	7.34	0.41	12.48
Kashkak	0.56	6.57	5.88	1.17	12.657	Asad Abad	0.25	7.76	7.81	2.78	12.27
Kameh Sofla	0.56	6.73	6.13	1.29	12.664	Heshmat Abad	0.60	7.11	7.21	2.71	12.64
Kameh Olia	0.36	7.05	6.39	0.81	12.446	Sarhang	0.22	7.87	8.03	1.97	12.25
Sorkhabad	0.47	6.62	5.54	0.78	12.567	Kal Qari	0.35	7.49	7.17	0.94	12.39
Khoroshbar	0.40	6.44	5.06	0.66	12.499	Pish Akhor	0.52	7.06	6.61	2.17	12.61
Besk	0.44	6.68	5.64	0.19	12.517	Soltan Abad	0.30	7.67	7.65	2.16	12.35
Fadihah	0.58	6.62	5.80	0.39	12.661	Asfiukh	0.18	7.46	6.94	2.44	12.25
Rud Majan	0.35	6.87	5.87	0.48	12.430	Robat Miandasht	0.03	8.02	8.01	3.64	12.17
Deh Paeen	0.45	6.86	6.10	0.89	12.464	Nasar	0.16	7.71	7.42	3.17	12.27
Fahandar	0.39	7.11	6.59	1.77	12.451	Mahmoud Abad	0.15	7.81	7.39	0.72	12.26
Khorram	0.66	6.93	6.87	4.47	12.736	Asad Abad	0.06	7.89	7.68	3.22	12.19
Deh Menar	0.37	7.71	7.78	1.17	12.402	Pangi	0.13	7.84	7.69	0.56	12.12
Abd Abad	0.41	7.39	7.34	2.28	12.436	Esfiz	− 0.08	8.10	8.10	2.16	11.91
Bors	0.22	7.37	6.92	1.73	12.268	Razg	0.47	7.02	6.55	0.11	12.60
Tajrud	0.48	7.18	7.03	2.61	12.446	Nasar	− 0.007	8.21	8.35	0.32	11.97
Houze Sorkh	0.36	7.12	6.51	3.20	12.388	Derakht Senjed	0.10	8.10	7.97	1.29	12.14
Seyuki	0.40	7.20	6.69	2.18	12.409	Nouri	0.52	7.28	7.39	2.48	12.53
Bouri Abad	0.46	7.23	6.94	3.19	12.481	Sarbala	0.27	7.73	7.51	1.16	12.28
						Nosrat Abad	− 0.38	8.71	8.31	1.28	11.82

## 2 Experimental design, materials and methods

### 2.1 Study area description

Torbat-e-Heydarie is one of the cities of Khorasan-e- Razavi province with an area of 3900 square kilometers located on 152 km south of the Mashhad city. The city with a population of 267,604 people and an area of 62220 square kilometers, between the meridian of 58 degrees and 41 min to 60 degrees 7 min east longitude and circuits of 34 degrees and 59 min to 35 degrees 51 min north latitude and its height from sea level free is 1333 m. Torbat-e-Heydarieis bordered from north with cities of Nishabur, Mashhad, fariman, from the East with the cities of Torbat Jam, Tayabad and Khaaf, from the south with the cities of Roshtkhar and Mahvelat and from the West with the city of Kashmar. The city has four parts: the central part, Jolgerokh, Kadkan and Bayag and also has 6 towns and 11 rural districts and 250 inhabited villages. There is no permanent major surface flow in the Torbat-e-Heydarie basin.

### 2.2 Materials and methods

In this cross-sectional study 188 samples from 47 water sources were taken, 18 parameters were evaluated according to the standard method, also in terms of Standard compliance were compared with Iran and EPA water standards [13], [14], [15], [16], [17]. Experiments conducted in two general categories of device experiments and Titration. Titration Experiment includes temporary and permanent hardness, magnesium, calcium and chlorides, Device Experiment consist of pH, Electrical conductivity, Turbidity, Fluorine, nitrate and sulfate [2], [18], [19], [20], [21]. Data were analyzed by using Excel software and descriptive statistics such as minimum, maximum, mean and standard deviation. Water stability statuses in rural area of Torbat-e-Heydarie were investigated by using Langelier Saturation, Ryznar Stability, Puchorius scale, Larson-Skold and Aggressive Index. All these parameters are listed summarized in Table 1.
